# Bias‐Polarity‐Dependent Direct and Inverted Marcus Charge Transport Affecting Rectification in a Redox‐Active Molecular Junction

**DOI:** 10.1002/advs.202100055

**Published:** 2021-06-19

**Authors:** Yingmei Han, Cameron Nickle, Maria Serena Maglione, Senthil Kumar Karuppannan, Javier Casado‐Montenegro, Dong‐Chen Qi, Xiaoping Chen, Anton Tadich, Bruce Cowie, Marta Mas‐Torrent, Concepció Rovira, Jérôme Cornil, Jaume Veciana, Enrique del Barco, Christian A. Nijhuis

**Affiliations:** ^1^ Department of Chemistry National University of Singapore 3 Science Drive 3 Singapore 117543 Singapore; ^2^ Department of Physics University of Central Florida Orlando FL 32816 USA; ^3^ Institut de Ciència de Materials de Barcelona (ICMAB‐CSIC)/CIBER‐BBN Campus de la UAB Bellaterra 08193 Spain; ^4^ Centre for Materials Science School of Chemistry and Physics Queensland University of Technology Brisbane Queensland 4001 Australia; ^5^ Australian Synchrotron Clayton Victoria 3168 Australia; ^6^ Laboratory for Chemistry of Novel Materials University of Mons Place du Parc 20 Mons B‐7000 Belgium; ^7^ Centre for Advanced 2D Materials and Graphene Research Center National University of Singapore 6 Science Drive 2 Singapore 117546 Singapore; ^8^ Hybrid Materials for Opto‐Electronics Group Department of Molecules and Materials MESA+ Institute for Nanotechnology and Center for Brain‐Inspired Nano Systems Faculty of Science and Technology University of Twente P.O. Box 217 Enschede AE 7500 The Netherlands

**Keywords:** charge transport, inverted Marcus region, molecular diodes, molecular tunnel junctions, self‐assembly

## Abstract

This paper describes the transition from the normal to inverted Marcus region in solid‐state tunnel junctions consisting of self‐assembled monolayers of benzotetrathiafulvalene (BTTF), and how this transition determines the performance of a molecular diode. Temperature‐dependent normalized differential conductance analyses indicate the participation of the HOMO (highest occupied molecular orbital) at large negative bias, which follows typical thermally activated hopping behavior associated with the normal Marcus regime. In contrast, hopping involving the HOMO dominates the mechanism of charge transport at positive bias, yet it is nearly activationless indicating the junction operates in the inverted Marcus region. Thus, within the same junction it is possible to switch between Marcus and inverted Marcus regimes by changing the bias polarity. Consequently, the current only decreases with decreasing temperature at negative bias when hopping is “frozen out,” but not at positive bias resulting in a 30‐fold increase in the molecular rectification efficiency. These results indicate that the charge transport in the inverted Marcus region is readily accessible in junctions with redox molecules in the weak coupling regime and control over different hopping regimes can be used to improve junction performance.

## Graphical abstract

Herein, a 30‐fold improvement in the performance of a molecular diode is demonstrated by pushing the redox‐reaction into the Inverted Marcus region in a two‐terminal molecular junction. This system transits from normal to inverted Marcus region due to electrostatic gating of the charged state with respect to the neutral state of an electron donor in response to an applied electric field.

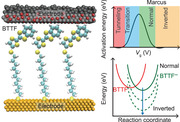

## 1 Introduction

Understanding the mechanisms of charge transport across molecules and molecule–electrode interfaces is important in countless areas of research,^[^ ^1^ ^]^ and in particular in molecular electronics to guide the design of efficient functional devices, such as, molecular diodes,^[^ ^2^ ^]^ molecular memory,^[^ ^3^ ^]^ and optoelectronic devices.^[^ ^4^ ^]^ Naturally, it is important to control the mechanism of charge transport between thermally activated and activationless charge transport regimes to ensure maximal performance of molecular junctions, but such control over charge transport regimes to improve device performance has not yet been demonstrated. The mechanisms of charge transport/transfer across molecules are often described using either one of the two extreme cases: Landauer theory which describes (essentially) temperature‐independent coherent tunneling in solid‐state junctions, or Marcus theory which describes thermally activated incoherent tunneling (which is also called hopping) under wet electrochemical conditions.^[^ ^5^ ^]^ There are quite a few examples of molecular junctions, however, whose charge transport characteristics cannot be straightforwardly explained with either theory, involving anomalous temperature dependencies, unusually small tunneling decay coefficients, or long‐range tunneling phenomena.^[^ ^1^, ^6^ ^]^ Here, we report a 30‐fold improvement in the performance of a molecular diode by switching from charge transport in the normal Marcus to the inverted Marcus region by changing the bias polarity. As a result, the activation barrier in inverted Marcus region at forward bias is eliminated, while at reverse bias thermally activated hopping can be frozen out by lowering the ambient temperature. These findings are important as they demonstrate how molecular–electronic devices can be improved by eliminating leakage currents and energy barriers (i.e., activation energy) for hopping.

**Figure 1:**
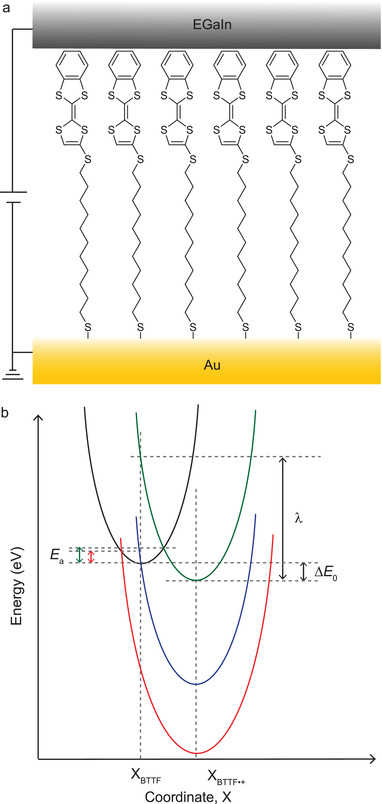
a) Schematic illustration of the Au‐S(CH_2_)_11_S‐BTTF//GaO*_x_*/EGaIn junction where EGaIn = eutectic alloy of Ga and In, GaO*_x_* is highly conductive and 0.7 nm thick, and “//” indicates the van der Waals contact.^[^ ^7^ ^]^ Here, the bottom gold electrode is grounded and the bias is applied to top EGaIn electrode. b) Nonadiabatic Marcus parabolas for different redox reaction energies as explained in the text.

The molecular diode is based on a self‐assembled monolayer (SAM) of benzotetrathiafulvalene derivative (BTTF; **Figure** **1**) which can be pushed into the activationless inverted Marcus region for hopping in one bias polarity, but not in the other, directly affecting its diode performance in terms of the rectification ratio *R = J*(*+V*)/*J*(−*V*) where *J* = current density and *V* = applied voltage. This results in a complex temperature‐dependent rectification behavior with the best diode performance at low temperature (*T* = 170 K) with *R* = 124 (30 times improvement over *R* at *T* = 320 K) when the leakage current at negative bias, i.e., *J*(−*V*), via hopping in the normal Marcus region is “frozen out.” In contrast, the diode remains “on” due to the activationless nature of hopping in the inverted Marcus region at positive bias, i.e., *J*(+*V*). Our results highlight the unique opportunity molecular junctions offer to study charge transport in the, otherwise difficult to access, inverted Marcus region.

In coherent tunneling, *J* decays exponentially defined by the tunneling decay coefficient *β* as a function of the tunneling distance *d*. In this regime, charge transport is essentially independent of *T*, and the general tunneling equation (Equation (1)) applies where *J*_0_ is a pre‐exponential factor

(1) $$J=J_{0}e^{-\beta d}$$

In the case of hopping, *J* depends on *T*, and the current through the molecule is described by the Arrhenius equation (Equation (2))

(2) $$J\;=J_{0}\;e^{-E_{a}/k_{B}T}$$

where *E*_a_ is the activation energy and *k*_B_ is the Boltzmann constant. In this regime, the current also depends on *d* but with significantly smaller *β* than that of coherent tunneling.^[^ ^5^, ^8^ ^]^ Figure 1b graphically illustrates the Marcus parabolas for charge transfer (or hopping) for an exothermic redox process where the black/green parabola depicts the potential energy curves of the donor–acceptor pair consisting of neutral molecule (here BTTF)/charged molecule (here BTTF^·+^).^[^ ^6^, ^9^ ^]^ The *E*_a_ is defined by the crossing between the two parabolas, representing the energy that must be overcome to enable charge hopping, Δ*E*_0_ is the energy difference between neutral and charged states, and *λ* is the molecular reorganization energy. The blue parabola shows a scenario in which the crossing point between both parabolas lies at the minimum potential eliminating *E*_a_ leading to temperature‐independent charge transport; this is the transition point into the inverted Marcus region beyond which an activation barrier is generally re‐established again, as given by red parabola and double arrow, respectively. As discussed in more detail below, in molecular junctions, however, due to the availability of electronic states in the electrodes, the charge transport in the inverted Marcus region remains activationless.^[^ ^6g^ ^]^

Although Marcus predicted the inverted Marcus region in 1956,^[^ ^10^ ^]^ it was not until 1984 the inverted Marcus region was experimentally proven^[^ ^11^ ^]^ because the electron transfer rate was much larger than the diffusion rate of the reactants in solution and, consequently, the charge transfer rate was diffusion limited.^[^ ^12^ ^]^ By using donor–bridge–acceptor (D‐b‐A) compounds in solution, the charge transfer reaction between D and A could be pushed into the inverted Marcus region because of the distance between D and A was fixed by a rigid spacer eliminating the diffusion limit.^[^ ^11^ ^]^ In contrast, in molecular tunnel junctions, the charge transfer across the molecules is studied in the solid state, and the molecules are connected to the electrodes: molecular tunnel junctions are therefore not diffusion limited and should in principle be good test beds to study charge transport in the inverted Marcus region. However, only recently we reported a solid‐state molecular junction with a D‐b‐A molecule that can be pushed from the normal to the inverted Marcus region via intramolecular orbital gating (which requires chemical modifications to the molecule to control intramolecular orbital gating)^[^ ^7^ ^]^ (since then a second example has been reported in a thin organic film device based on hot‐electron injection^[^ ^13^ ^]^ and a third example based on a bipyridyl molecular junction where the authors propose that a gating effect induced by Fermi level pining could push the junction in the Marcus inverted region^[^ ^14^ ^]^). Here, we demonstrate junctions with only electron donor units (which have a much simpler structure than D‐b‐A units) can be pushed into the inverted Marcus region via gating by the applied electric field because the BTTF in the neutral state only responds weakly, while the cationic BTTF^•+^ unit responds strongly, to changes in the applied electric field.

## 2 Results and Discussion

### 2.1 Monolayer Characterization

The SAMs of S(CH_2_)_11_S‐BTTF on Au were prepared with a previously reported procedure (Section S1, Supporting Information). The cyclic voltammogram (CV) of S(CH_2_)_11_S‐BTTF SAM on Au (Section S2, Supporting Information) shows two pairs of well‐resolved redox peaks at *E*_pa_/*E*_pc_ = +0.51 V/+0.53 V and +0.87 V/+0.89 V which corresponds to the oxidation of BTTF unit to radical cation (BTTF^•+^) and dication (BTTF^2+^). The fact that the full‐width‐at‐half maximum (FWHM) of the second oxidation peak is (76 mV) smaller than that of the first oxidation peak (146 mV) indicates that the SAMs are well‐ordered and densely packed.^[^ ^15^ ^]^ With photoelectron and X‐ray absorption spectroscopy, we determined the energy of the lowest unoccupied molecular orbital (*E*_LUMO_), highest occupied molecular orbital (*E*_HOMO_), and HOMO‐1 (*E*_HOMO‐1_) as described in Section S2 in the Supporting Information. The offset in energy between *E*_LUMO_ and the Fermi level (*E*_F_) of the electrode (*δE*_LUMO_) is 2.17 eV, and the energy offsets between *E*_HOMO_ and *E*_HOMO‐1_ and *E*_F_ (*δE*_HOMO_ and *δE*_HOMO‐1_) are 0.44 and 2.09 eV, from which we conclude that the HOMO is energetically accessible in the applied bias window.

### 2.2 Temperature‐Dependent Rectification

**Figure 2:**
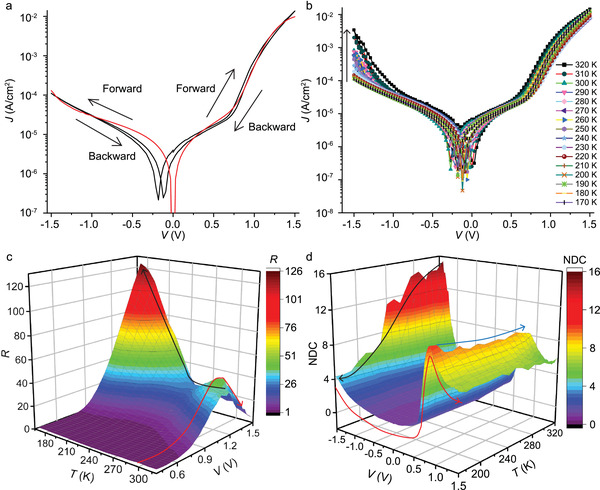
a) Representative *J*(*V*) curve of Au‐S(CH_2_)_11_S‐BTTF//GaO*_x_*/EGaIn junction recorded at *T* = 170 K (black line). The black arrows indicate the voltage scan direction. The red line is the fit to the single level model. b) Temperature‐dependent *J*(*V*) curves from 170 to 320 K at intervals of 10 K. The black arrow indicates the increase of *J* with *T*. c) 3D surface with color map of *R* plotted against *V* and *T* and d) of the corresponding NDC plots using the *J*(*V*) curves shown in panel (b). The red and black arrows in panel (c) indicate how *R* changes with *V* and *T*, respectively. The blue and black arrows in panel (d) indicate how the HOMO peaks evolve with *T* at positive and negative bias, respectively. The red arrow indicates how NDC curves evolve with *V* at low temperature (see Figure S5 in the Supporting Information for the corresponding 2D plots).

We performed *J*(*V*,*T*) measurements using a top electrode of EGaIn stabilized in a microchannel in polydimethylsiloxane aligned over micropores (with an area of 3.1 × 10^2^ µm^2^) in 10 nm thick AlO*_x_* on template‐stripped Au as reported in ref. [16] (Section S3, Supporting Information). We only selected junctions that had their *J*(*V*) characteristics within one Gaussian log‐standard deviation of the <log|*J*|>_G_ versus *V* curve, which is reported in ref. [17], to perform the *J*(*V*,*T*) measurement (Section S4, Supporting Information). **Figure** **2**a shows a *J*(*V*) characteristic of the Au‐S(CH_2_)_11_S‐BTTF//GaO*_x_*/EGaIn junction measured at *T* = 170 K and that this junction rectifies at positive bias with a rectification ratio *R =* 124 with *R* defined by Equation (3)

(3) $$R\equiv J\left(+1.5\;V\right)/\left|J\left(-1.5V\right)\right|$$

where *J*(−1.5 V) is defined as the leakage current^[^ ^18^ ^]^ that flows across the diode in the off‐state. The junctions show a small, but significant, hysteresis. A similar hysteresis has also been observed for molecular diodes of the form of Ag‐S(CH_2_)_11_Fc//GaO*_x_*/EGaIn (Fc is ferrocene).^[^ ^19^ ^]^ This hysteresis was associated with charging of the Fc unit to Fc^+^ at forward bias and discharging of the Fc^+^ units back to Fc at reverse bias, probably stabilized by the GaO*_x_* layer. Therefore, we believe that the small hysteresis is caused by (dis)charging of the BTTF or BTTF^·+^ units. Here, we note that despite the similar redox properties of BTTF and Fc, the direction of rectification is reversed for these two types of molecular diodes. We attribute this reversal of the rectification to the different coupling strength between the redox units and EGaIn top electrode where the BTTF units interact much weakly with the EGaIn top electrode (see figure 7 of ref. [17]) than the Fc units (see figure 8 of ref. [19]). Besides, a small current near 0 V was observed, which can be caused by the capacitive behavior of the junctions, or involve the GaO*_x_* layer as we have explained in previous work.^[^ ^20^ ^]^

Figure 2b shows the *J*(*V*,*T*) curves in the temperature range of 170–320 K. Close to room temperature (RT), at large negative applied bias a clear upturn of the current is visible which indicates that the HOMO enters the conduction window, but with decreasing *T* conduction through this orbital at −1.5 V decreases by a factor of 31 (indicated by the black arrow) which is characteristic for hopping (see below). In contrast, conduction through the HOMO at +1.5 V positive bias depends only weakly on *T*. This decrease in the current is the reason why *R* is large at low *T*, but small at RT, as reduction of *T* only lowers the leakage current—the current that flows at −1.5 V when the diode is in the off‐state—and not the current that flows across the diode in the on‐state at +1.5 V (Equation (3)). Figure 2c shows the 3D color map of *R*(*V*,*T*) and that at RT the *R*(*V*) goes through a maximum value because first *R* increases when the HOMO enters the conduction window at relatively small positive bias, but *R* decreases again when the HOMO enters the conduction window at relatively large negative bias (as indicated by the red arrow).

### 2.3 Modeling of the Diode Behavior

We have modeled the current through the junction with the standard Landauer theory for a single level tunnel junction using the following expressions^[^ ^5c^ ^]^

(4) $$\begin{array}{c} I=n\frac{q}{h}\int \int_{-\infty }^{\infty }dEdE^{\prime }D_{E^{\prime }}\left(E\right)G_{\varepsilon }\left(E^{\prime }\right)\frac{\gamma_{L}\gamma_{R}}{\gamma_{L}+\gamma_{R}}\left[f_{L}\left(E\right)-f_{R}\left(E\right)\right]\\ D_{E^{\prime }}\;\left(E\right)=\frac{\gamma /2\pi }{{\left(E-\left(E^{\prime }+\left(\eta -\frac{1}{2}\right)V\right)\right)}^{2}+{\left(\gamma /2\right)}^{2}}\\ G_{\varepsilon }\left(E^{\prime }\right)=\;\exp\left(\frac{{\left(E^{\prime }-\varepsilon \right)}^{2}}{2\sigma^{2}}\right) \end{array}$$

where *n* is the number of molecules contributing to conduction, *γ*_*L*_ and *γ*_*R*_ are the tunneling rates between the molecule and the left (the EGaIn top electrode) and right (the Au bottom electrode) electrodes (respectively), *D*_*E*′_(*E*) is the electronic density of states of the molecular level (represented by a normalized Lorentzian function centered at energy $E^{\prime }+\left(\eta -\frac{1}{2}\right)V$ and width *γ*  = *γ*_L_  + *γ*_R_) with its connection to the electrodes represented by the voltage division parameter *η*  = *V*_R_ /(*V*_L_ + *V*_R_), *G*_*ε*_(*E*′) is a Gaussian function (with center *ε* and width *σ*) that represents the inherent dispersion of the molecular level energy in an ensemble of molecules (rather than a single‐molecule junction), and *f*_L_(*E*) and *f*_R_(*E*) are the Fermi functions representing the electronic occupation of the left and right electrodes, respectively (see for details ref. ^[5c^ ^]^). A good fitting to the data (Figure 2a) is achieved for *T* = 170 K and the following set of parameters: $\varepsilon \equiv \delta E_{\mathrm{HOMO}}^{\mathrm{th}}/e\;=\;0.83\;V$ (zero‐bias energy of the molecular orbital with respect to the Fermi energy of the electrodes, due to the charge transfer between BTTF molecule and top EGaIn electrode, the HOMO level downshift by 0.4 eV compared to that measured for SAMs^[^ ^17^ ^]^ (Table S2, Supporting Information)), *n* = 150, *γ*_L_ =  9.92  × 10^−3^ eV, *γ*_R_ =  9.88  × 10^−7^ eV, *η*  =  0.37, and *σ*  =  0.11 eV. As demonstrated in Figure 1a, the BTTF units are separated from the Au electrode by the long alkyl chains but are in van der Waals contact with top EGaIn electrode, resulting in *γ*_L_ ≫ *γ*_R_. As can be extracted from Equation (4), the overall tunneling rate in the junction is given by $\Gamma =\frac{\gamma_{L}\gamma_{R}}{\gamma_{L}+\gamma_{R}}=9.88\times 10^{-7}\;\mathrm{eV}$, denoting a molecule weakly coupled to the electrodes. This analysis confirms that the HOMO dominates charge transport both bias polarities, but that it enters the bias window at different voltages which explains the mechanism of rectification.^[^ ^21^ ^]^

The complex interplay between *R* and tunneling involving the HOMO level at opposite bias polarities is confirmed by normalized differential conductance (NDC) analysis, where NDC = (*dI*/*dV*) * (*V*/*I*),^[^ ^16^, ^22^ ^]^ of the *J*(*V*) curves as a function of *T* shown in Figure 2d. NDC is similar to *dI*/*dV* analysis which is widely used in community to identify molecular resonances.^[^ ^22^, ^23^ ^]^ Therefore, here the resonance peak at +0.90 V with NDC = 9 indicates charge transport through the HOMO (red arrow), and the large NDC value is characteristic for diode behavior.^[^ ^22^ ^]^ Strikingly, this NDC peak is quite insensitive to changes in *T* (blue arrow). At negative bias, no peaks are visible to about −1.15 V as expected for off‐resonant tunneling, but at large negative bias the NDC value increases and a peak is visible at *V* = −1.36 V for *T* > 250 K confirming the HOMO enters the conduction window. The NDC value of this HOMO peak is highly temperature sensitive and decreases from 15 (at 320 K) to 4.5 (at 170 K) as indicated by the black arrow, which implies that hopping dominates the mechanism of charge transport at large negative bias. This “freezing out” of the hopping mechanism lowers the leakage currents resulting in an increase of *R* (the black arrow in Figure 2c). Such behavior where the temperature dependency of incoherent hopping changes within the same junction at different bias polarities has not been reported before.

### 2.4 Determination of Activation Energies

**Figure 3:**
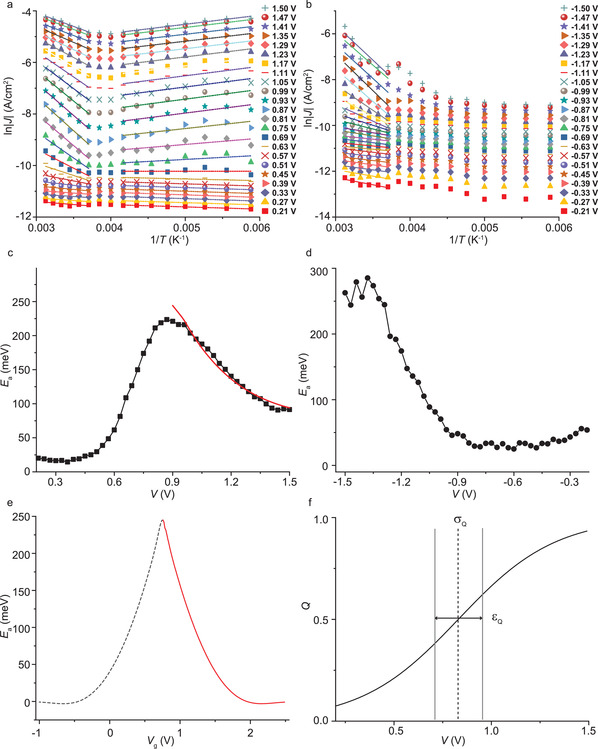
Arrhenius plots a) from 0.21 to 1.5 V with 60 mV intervals and b) from −0.21 to −1.5 V with −60 mV interval for the data set in Figure 2b (note due to capacitive background currents and noise at low bias, the data points from 0 to 0.21 V and 0 to −0.21 V were omitted from the modeling). The solid and dashed lines are fits to Equation (2) (see Figure S5 in the Supporting Information for the full dataset). Here, we note that some data points at high temperatures and at large applied bias suffer from noise likely because these conditions approach the breakdown voltage of our devices.^[^ ^31^ ^]^ c) *E*_a_ versus *V* at positive bias. The solid red line is a fit using the Marcus rates given in Equations (5a) and (5b). As explained in the main text, the increase in *E*_a_ originates from the HOMO entering the bias window, which indicates that the junction is not in the Marcus hopping regime until the maximum *E*_a_ was reached. Therefore, only half of the bell‐shaped curve was used (solid red line) to fit our data. d) *E*_a_ versus *V* at negative bias. e) Activation energy as a function of gate voltage according to Migliore's model using Marcus charge transfer rates in the hopping regime. We used the red solid line to fit the experimental data in panel (c). f) Charge distribution used for the fitting in panel (c).

To investigate the reason why conduction through the HOMO at +1.5 V depends only weakly on *T*, we determined the values of *E*_a_ as a function of positive applied bias. From the *J*(*V*) curves recorded at different temperatures (as shown in Figure 2b), we constructed the Arrhenius plots from which the *E*_a_ was extracted at temperatures above 260 K with Equation (2) for each applied voltage (**Figure** **3**). Figure 3c shows that the *E*_a_ plotted against *V* goes through a maximum at +0.87 V, which is very similar to the resonance peak observed in the NDC plots at +0.90 V. Therefore, we explain this bell‐shaped *E*_a_ versus *V* curve as follows. As soon as the HOMO enters the bias window, the mechanism of charge transport changes from (activationless) coherent off‐resonant tunneling to (temperature dependent) incoherent tunneling (as evident from the NDC analysis) resulting in an increase of *E*_a_.^[^ ^24^ ^]^ Due to the broadening of HOMO level, this transition of the system from the point when the HOMO starts to enter the conduction window to the point when the HOMO has fully entered the conduction window needs to go through a certain voltage range, resulting in the increase of *E*_a_ with applied bias until a maximum value of *E*_a_ = 224 meV at 0.87 V is reached. In principle, when the HOMO is close to the Fermi level of the electrode, coherent on‐resonant tunneling can occur provided that the molecule interacts strongly with the electrodes, i.e., strong coupling regime,^[^ ^25^ ^]^ or have unfavorable *λ* (and are, consequently, not redox active),^[^ ^26^ ^]^ which is not the case in our EGaIn junction since the redox units are separated from the bottom electrode by the alkyl chain and form a weak van der Waals contact with the top electrode where the thin GaO*_x_* layer weakens the molecule–top electrode interaction even further,^[^ ^16^ ^]^ and BTTF units are well‐known to be redox active. Remarkably, after reaching a maximum value, *E*_a_ decreases with increasing *V*. This behavior cannot be directly explained by coherent tunneling described by Landauer–Buttiker theory or hopping described by Marcus theory for the following reasons.^[^ ^6^, ^7^ ^]^ The small *E*_a_ in coherent tunneling originates from the Fermi‐level broadening which is very small (and often not observed in molecular tunnel junctions apart from a few exceptions^[^ ^24^, ^27^ ^]^) and decreases with increasing bias.^[^ ^5^, ^24^ ^]^ However, in this work, the observed *E*_a_ is larger than 200 meV and first increases and then decreases with the applied voltage. Second, the activation energy for hopping remains constant as a function of applied voltage (after an initial sharp increase when the molecular orbital enters the conduction window). On the other hand, although Poole–Frenkel transport and thermionic emission also result in a voltage‐dependent activation energy, they predict a linear relationship between *E*_a_ and *V* ^1/2^, which is not the case in this system.^[^ ^6c^ ^]^ In the following section, we will explain this behavior in more detail by using the combined model proposed by Migliore et al.^[^ ^6g^ ^]^ which reproduces the bell‐shaped *E*_a_ versus *V* curve well. In contrast, EGaIn junctions lacking redox‐active units,^[^ ^16^, ^28^ ^]^ the bell‐shaped *E*_a_ versus *V* curve is not observed, which demonstrates that this transition is unique to the redox‐active molecular junctions. At negative bias, *E*_a_ increases with increasing bias when the HOMO enters the conduction window. Here, the downturn of *E*_a_ is not visible because the junctions are not stable at an applied bias larger than −1.5 V. Interestingly, in the temperature range of 220–130 K, a small but reproducible negative *E*_a_ (−30 meV for *V* = 1.5 V) is observed (Figure 3a). It is well known that TTF^•+^ dimerizes.^[^ ^29^ ^]^ Schröder et al.^[^ ^29c^ ^]^ have demonstrated that TTF^•+^ is the dominant species at ambient temperatures, but the fraction of the dimer increases with decreasing temperature. This dimerization could account for the observed negative *E*_a_ in our junctions as follows. At high temperatures, there is large fraction of BTTF^•+^ monomers and charge transport predominantly proceeds via these monomers. With increasingly low temperatures, more and more dimers form through which then charge transport occurs. We have shown before that for viologen dimers (whose radical form also forms dimers) the charge transport rates are about 100 times larger than for the monomers.^[^ ^3a^ ^]^ Therefore, the current across the junctions may increase with decreasing temperature because of dimer formation across which tunneling is more efficient than across the monomers, resulting in an apparent negative *E*_a_. Alternatively, a temperature‐dependent change in the molecule–electrode coupling could possibly account for the negative *E*_a_.^[^ ^30^ ^]^

### 2.5 Charge Transport in the Inverted Marcus Region

**Figure 4:**
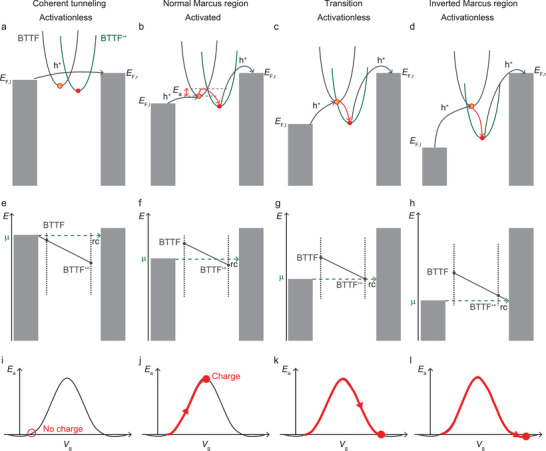
a–d) Marcus parabolas of the BTTF→BTTF^·+^ redox reaction inside the junction for different applied bias to the left electrode. The black arrows indicate charge transport between the electrodes and the BTTF unit. The red arrows indicate the transition between BTTF and BTTF^·+^. e–h) The corresponding redox reaction depicted on a reaction coordinate (rc) with respect to *μ*. i–l) The *E*_a_ versus *V*_g_ where the red dots correspond to the *E*_a_ in different charge transport regimes as sketched in panels (a)–(d).

To explain the data in Figure 3c, we have used the model developed by Migliore et al.^[^ ^6g^ ^]^ to fit our data (Section S5, Supporting Information) and **Figure** **4** summarizes the mechanism of charge transport explaining the observed *E*_a_(*V*) curve. In this model, the rates of the charge transfer from the neutral BTTF and charged BTTF^·+^ states are given by Equations (5a) and (5b)

(5a) $$K_{\mathrm{BTTF}\to \mathrm{BTT}F^{\cdot +}}=\frac{1}{\sqrt{4\pi \lambda k_{B}T}}\int dE\Gamma \left(E\right)e^{-\frac{{\left(\Delta E+E-\lambda \right)}^{2}}{4\lambda k_{B}T}}f\left(E\right)$$

(5b) $$K_{\mathrm{BTT}F^{\cdot +}\to \mathrm{BTTF}}=\frac{1}{\sqrt{4\pi \lambda k_{B}T}}\int dE\Gamma \left(E\right)e^{-\frac{{\left(\Delta E+E-\lambda \right)}^{2}}{4\lambda k_{B}T}}\left[1-f\left(E\right)\right]$$

here Γ(*E*) is the golden rule rate for electron transfer between the single electron level on the molecule and single electron states of energy *ε* on the metal, *f*(*E*) is the Fermi distribution of the electron occupation in the electrodes which accounts for a weak temperature dependence via Fermi level broadening, Δ*E*  =  Δ*E*_0_ + *μ*, where $\Delta E_{0}=\delta E_{\mathrm{BTT}F^{\cdot +}}^{\mathrm{th}}-\delta E_{\mathrm{BTTF}}^{\mathrm{th}}$ is the energy difference between the two molecular charge states (BTTF and BTTF^·+^ in our case) at zero bias and *μ* is the electrochemical potential of the electrodes. According to the model by Migliori et al.,^[^ ^6g^ ^]^ a gating electric potential changing the relative energy difference (Δ*E*(*V*_g_)) between the two charge states (i.e., affecting one orbital differently than the other and shifting the energy of the corresponding Marcus parabolas with respect to each other), would lead to a gate‐dependent activation energy that can be calculated as *E*_a_/*k*_B_ *T*  =  *T*∂ln(*R*_Marcus_)/∂*T* (where *R*_Marcus_ is the sum of the two Marcus rates in Equations (5a) and (5b)). This *E*_a_ versus *V*_g_ dependence is shown in Figure 3e. As discussed above, we used half of the bell shape (red solid line) to calculate the parameters used below to fit the experimental data in Figure 3a, where the gate voltage is taken proportional to the charge in the molecule: $\;V_{g}=\;Q\left(V\right)/C_{C}^{*}$, with *Q* increasing with bias and where $C_{C}^{*}$ is a fitting parameter that represents the capacitive coupling strength between the molecule and the electrode (responsible for the gating effect of BTTF^·+^). To be more specific, the change in *E*_a_ is induced by shifting the Marcus parabolas with respect to each other because the different charge states of BTTF molecule interact differently with the applied electric field: BTTF^·+^ interacts very strongly with the applied field (because of its charge) in contrast to neutral BTTF which can only interact weakly with the applied field. Likely, intermolecular interactions (e.g., *π*–*π* interactions, dimer formation, or polarization effect via induced dipoles) between neighboring BTTF^•+^ units likely also help to stabilize the charge on the molecules.^[^ ^3^, ^32^ ^]^

The activation energy expected from this model only applies when the transport through the molecule is through hopping, which only occurs for sufficiently large bias. At low applied bias (Figure 4a), the HOMO is not energetically accessible because *μ* lies above the energy of BTTF and BTTF^·+^ states in equilibrium with the environment. Figure 4e shows the transition from BTTF and BTTF^·+^ on the reaction coordinate (rc) which is represented by the dashed green line (not to be confused with distance). The bottom row shows the evolution of *E*_a_ as a function of the “gate voltage,” *V*_g_, which increases with bias and changes *E*_0_ (as defined in Figure 1). In this situation, the mechanism of charge transport across the junction is temperature independent off‐resonant coherent tunneling. As discussed above, *E*_a_ increases with increasing bias when the HOMO starts to enter the conduction window. *E*_a_ reaches a maximum value when HOMO fully enters the conduction window (Figure 4j). Now the redox reaction BTTF→BTTF^·+^ becomes important and the mechanism of charge transport changes to thermally activated incoherent hopping (Figure 4b). Thus, from this point onward, the junction enters the normal Marcus region when *μ* lies between BTTF and BTTF^·+^ on the reaction coordinate (Figure 4f) and *E*_a_ decreases with increasing bias. With increasing applied *V*, the BTTF^·+^ state is stabilized by the increasing electric field and therefore lowered in energy with respect to the parabola of the neutral state (which is essentially nonresponsive to the changes in the electric field because it is charge neutral). This “gating” effect of BTTF^·+^ increases *E*_0_ until one of the parabolas intersect the minimum of the other (Figure 4c,g,k) at which point *E*_a_ decreases to 0 eV. Beyond this point, increasing *V* would further force the junction into the inverted Marcus region (Figure 4d) and *E*_a_ should increase again (as indicated by the red arrows in Figure 1b). However, this does not happen in metal‐molecule‐metal junctions because the continuous availability of electronic states below the Fermi energy of the electrodes would pin the system to the minimum of the BTTF Marcus parabola (Figure 4h,i), resulting in activationless charge transport for any larger bias.

Figure 3f shows the *Q*(*V*) functional that was used as a result of the electronic current through the junction, where its inflection point ($\varepsilon_{Q}\equiv \delta E_{\mathrm{HOMO}}^{\mathrm{th}}/e$) and width (*σ*_*Q*_) of the charging curve are also fitting parameters constrained by the experimental *J*(*V*) curves ($\varepsilon_{Q}∼\varepsilon \equiv \delta E_{\mathrm{HOMO}}^{\mathrm{th}}/e$ and $\sigma_{Q}∼\sigma$). The model fits well to the data resulting in *λ*  =  1.20 eV, Δ*E*_0_ =  0.75 eV, *ε*_*Q*_ =  0.83 V(  =  *ε*), $\sigma_{Q}=\;0.25\;\mathrm{eV}\left(∼\sigma \right)$, and $C_{C}^{*}=$ 1.31 ×  10^−19^ F, accounting for the decrease in activation energy and proving that the junction at large positive bias moves toward the inverted Marcus regime. This model implies that the energy of the charged BTTF^·+^ state is highly stabilized by the applied bias resulting in large shifts (i.e., the maximum gate potential obtained when the orbital is fully charged, *Q* (*V*  =  1.5 V) =  *e* is $\;V_{g}^{\max}=\;e/C_{C}^{*}=\;1.18$ V) with respect to the energy of the neutral state (i.e., Δ*E*_0_), which is conceivable given the large electric fields on the order of *GV*/*m* typically encountered in molecular junctions. Although a clear maximum in the *E*_a_ versus *V* plot followed by a decrease in *E*_a_ are observed in all junctions (Figure 3c and Figure S8, Supporting Information), we note that a small *E*_a_ is observable at very small applied bias and also at large positive bias. This “background” *E*_a_ cannot be caused by hopping involving molecular orbitals since none fall in the bias window at low bias, but it may involve conformational changes^[^ ^29a,29b^ ^]^ or thermal broadening of the Fermi level of the electrode.^[^ ^5c^ ^]^ At large positive bias, dimer formation (it is well‐known that TTF radical cations can dimerize^[^ ^33^ ^]^ with an activation energy of several hundred meV^[^ ^34^ ^]^) could explain the observed *E*_a_.

## 3 Conclusions

In summary, our results show that complex temperature‐dependent behavior—involving bias polarity‐dependent Marcus and inverted Marcus regions—can be readily observed in molecular tunneling junctions because molecular junctions are not diffusion limited. Thus, the Inverted Marcus region is not only limited to D‐b‐A molecules where the energy of the HOMO can be shifted with respect to the LUMO via intramolecular orbital gating^[^ ^7^ ^]^ and can be accessed in other types of redox molecules only consisting of donor units as we show here. To obtain large enough “gating” voltages resulting in the changes in Δ*E*_0_ needed to push the system into the inverted Marcus region, charges have to relax on the molecule and, consequently, junctions in the weak coupling regime with redox‐active molecules are good candidates to observe this kind of complex temperature‐dependent behavior. As mentioned above, the low value of Γ = 9.88 × 10^−7^ eV indicates that our junctions operate in the weak coupling limit. The coupling between the BTTF and EGaIn top electrode is weak (compared to junctions in the strong coupling regime with chemisorbed species) because of the physisorbed nature of this contact and the presence of the 0.7–1.0 nm thick GaO*_x_* layer and the –(CH_2_)_11_– alkyl chain decouples the BTTF units from the bottom electrode. This explains why Marcus inverted region has so far been only rarely observed in solid‐state junctions because usually molecular junctions operate in the strong coupling regime (especially for single‐molecule junctions) and intramolecular orbital gating voltage was not high enough. Our results suggest that the inverted Marcus region may be readily accessible in other junction platforms and provide guidelines for future experiments to explore and investigate complex temperature‐dependent charge transport phenomena in general. We also note that although the inverted Marcus region is reached in both BTTF and bipyridyl^[^ ^14^ ^]^ based molecular junctions, the dependence of the rectification behavior on the temperature is different: for BTTF‐based molecular junctions, the rectification ratio increases (by a factor of 30) with decreasing temperature, while in the bipyridyl‐based molecular junctions, the rectification ratio does not depend on the temperature. It also would be very interesting to extend our studies to single‐molecule transistors where it is in principle possible to study the mechanisms of charge transport as function of the oxidation state of the BTTF unit.

## Conflict of Interest

The authors declare no conflict of interest.

## Supporting information

Supporting Information

## Data Availability

The data that support the findings of this study are available in the Supporting Information of this article.
