# Supplementary material for: Bias‐Polarity‐Dependent Direct and Inverted Marcus Charge Transport Affecting Rectification in a Redox‐Active Molecular Junction
Source: Adv Sci (Weinh). 2021 Jun 19;8(14):2100055. doi: 10.1002/advs.202100055 (PMC8292891; doi:10.1002/advs.202100055)
Supplement: Supplementary file 1 — Supporting Information [file ADVS-8-2100055-s001.pdf]

© 2021 Wiley-VCH GmbH

ADVANCED SCIENCE
Open Access

Supporting Information

for *Adv. Sci.*, DOI: 10.1002/advs.202100055

Bias-Polarity Dependent Direct and Inverted Marcus Charge Transport Affecting Rectification in a Redox-Active Molecular Junction

Yingmei Han,[1] Cameron Nickle,[2] Maria Serena Maglione,[3] Senthil Kumar Karuppannan,[1] Javier Casado-Montenegro,[3] Dongchen Qi,[4] Xiaoping Chen,[1] Anton Tadich,[5] Bruce Cowie,[5] Marta Mas-Torrent,[3] Concepció Rovira,[3] Jérôme Cornil,[6] Jaume Veciana,[3] Enrique Barco,[2*] and Christian A. Nijhuis[1,7,8*]*

Supplementary Information

Bias-Polarity Dependent Direct and Inverted Marcus Charge Transport in a Redox-Active Molecular Junction

Yingmei Han,[1¶] *Cameron Nickle,*[2¶] *Maria Serena Maglione,*[3¶] *Senthil Kumar Karuppannan,*[1¶] *Javier Casado-Montenegro,*[3] Dongchen Qi,[4] *Xiaoping Chen,*[1] *Anton Tadich,*[5] *Bruce Cowie,*[5] *Marta Mas-Torrent,*[3] *Concepció Rovira,*[3] *Jérôme Cornil,*[6] *Jaume Veciana,*[3*] *Enrique Barco,*[2*] *and Christian A. Nijhuis*[1,7,8*]

[1]Department of Chemistry, National University of Singapore, 3 Science Drive 3, Singapore 11754, Singapore.

[2]Department of Physics, University of Central Florida, Orlando, Florida, United States.

[3]Institut de Ciència de Materials de Barcelona (ICMAB-CSIC)/CIBER-BBN, Campus de la UAB, 08193 Bellaterra, Spain.

[4]Centre for Materials Science, School of Chemistry and Physics, Queensland University of Technology, Brisbane, Queensland 4001, Australia.

[5]Australian Synchrotron Clayton, Victoria 3168, Australia.

[6]Laboratory for Chemistry of Novel Materials, University of Mons, Place du Parc 20, B-7000, Mons, Belgium.

[7]Centre for Advanced 2D Materials and Graphene Research Center, National University of Singapore, 6 Science Drive 2, Singapore 117546, Singapore.

[8]Hybrid Materials for Opto-Electronics Group, Department of Molecules and Materials, MESA+ Institute for Nanotechnology and Center for Brain-Inspired Nano Systems, Faculty of Science and Technology, University of Twente, 7500 AE Enschede, The Netherlands

[¶]Authors who contributed equally

*Authors to whom correspondence should be addressed: vecianaj@icmab.es, delbarco@physics.ucf.edu, and c.a.nijhuis@utwente.nl

Section S1. Fabrication of template stripped Au bottom electrode and self-assemble monolayer (SAM) formation. We followed a previously reported procedure to fabricate the template stripped Au bottom electrode.[1] Briefly, 200 nm Au film deposited on silicon (100) wafers by using thermal evaporator (Shen Yang Ke Yi, China), and then the thermal glue (EPOTEK 353ND) was used to glue clean glass slides on the Au surface. The Au/thermal glue/glass stacks were then subsequently cured at 80 °C for 3 hours, followed by removal of the metal-glue-support from the template. The freshly template stripped Au substrates were immersed in degassed 0.5 mM $(S(CH_2)_{11}S\text{-}BTTF)_2$ solution in toluene immediately to minimize the contamination from the ambient environment. After that, the solution was heated at 45° C for 3 hours and left untouched under N_2 atmosphere for ~21 hours. Finally, after the SAM formation, the substrates were washed with toluene and dried with a gentle stream of N_2.

Section S2. SAM characterization

Cyclic voltammetry. The $(S(CH_2)_{11}S\text{-}BTTF)_2$ SAM on Au was characterized by cyclic voltammetry. Silver and platinum wires were used as quasi-reference and counter electrodes, SAM on Au was used as a working electrode. We used a 0.1 M solution of $LiClO_4$ in acetonitrile as an electrolyte. The surface coverage (Γ) of BTTF unit was determined by following previously reported method[2]. Figure S1 shows the cyclic voltammogram (CV) of $S(CH_2)_{11}S\text{-}BTTF$ SAM on Au under different scan rates. The linear relationship between the peak current and scan rates demonstrated the $S(CH_2)_{11}S\text{-}BTTF$ is confined on the surface.

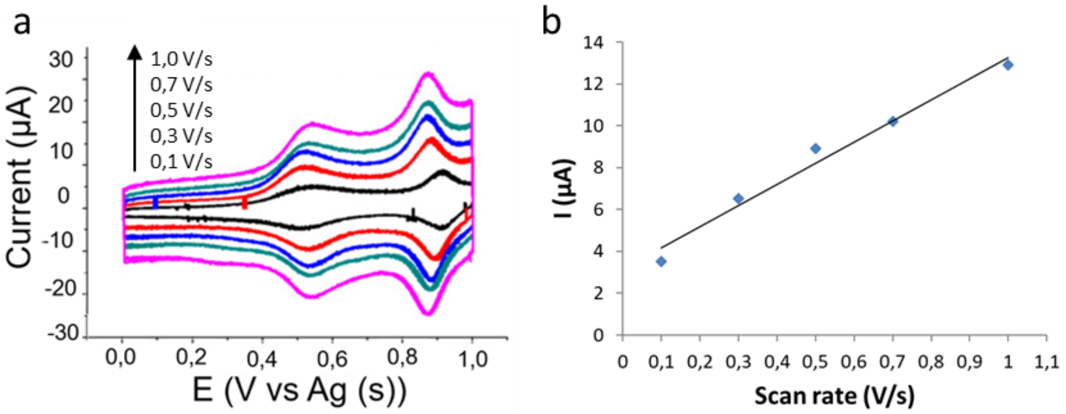

Figure S1. (a) CV of the $S(CH_2)_{11}S$-BTTF SAM on Au measured at different scan rates. (b) peak currents vs. scan rate.

Table S1. Supramolecular properties of $S(CH_2)_{11}S$-BTTF SAM on Au.

	CV	NEXAFS		ARXPS		
Metal	Γ(×10^{-10} mol/cm^2)	Tilt angle [°][a)]	d_{SAM} [Å][b)]	Elemental ratio		
				S_2:S_1 (theo)	C_1:C_2 (theo)	C:S (theo)
Au	1.4	58.5	22.5	6.1 (5.0)	1.3 (0.9)	16.2 (3.5)

[a)]The tilt angle was the average tilt angle of the BTTF unit with respect to the surface normal. The error of the tilt angle is ± 5°. The tilt angle of BTTF was defined as the angle between the BTTF plane and the surface normal.
[b)]The error in d_{SAM} is 5% from fitting error in peak areas.

Table S2. Electronic properties of $S(CH_2)_{11}S$-BTTF SAM on Au

	UPS[a)]					NEXAFS
Metal	WF_{BTTF} [eV]	δE_{HOMO} [eV]	E_{HOMO} [eV]	δE_{HOMO-1} [eV]	E_{HOMO-1} [eV]	E_{LUMO} [eV]
Au	4.48	0.44	-4.92	2.09	-6.57	-2.31

[a)] Resolution for UPS determination was ± 0.05 eV.

Angle-resolved X-ray photoelectron spectra (ARXPS) and near-edge X-ray absorption fine structure spectroscopy (NEXAFS). The ARXPS and NEXAFS spectra were recorded at Australian synchrotron by following previously reported procedures.[3] The measurements were conducted under the ultra-high vacuum of ~10^{-10} mbar at room temperature. ARXPS spectra were recorded at a take-off angle of 90°, and 20° and NEXAFS spectra were recorded at an incidence angle of 90° (NI) and 20°(GI).

We analyzed the ARXAPS S 2*p* and C 1*s* spectra by following previously reported procedure.[4] Figure S2a shows the S 2*p* spectra, which are dominated by two peaks (S_1 and S_2). S_1 at 162.0 eV is attributed to Au-S, and S_2 at 163.5 eV is attributed to the S that links the BTTF unit and the alky chain, the S in the BTTF unit and the physiosorbed S. The contribution of S_2 increase from take-off angle of 90° to 20°, and the contribution of S_1 decreased from 90° to 20°, indicating the standing up phase of the $S(CH_2)_{11}S$-BTTF. Figure S2b shows the C 1*s* spectra, which are dominated by two peaks (C_1 and C_2). C_1 at 284.8 eV is attributed to C=C, C_2 at 285.3 eV is attributed to C=C. The elemental ratio of S_2:S_1 is 6.1 and C_1:C_2 is 1.3, which is consistent with the calculated elemental ratio (Table S1). The SAM thickness was determined by comparing the S_1 peak intensities at a take-off angle of 90° and 20°, which followed a previously reported procedure.[4]

Figure S3 shows the NEXAFS spectra of $S(CH_2)_{11}S$-BTTF SAM on Au. We observed a resonance peak at 285.05 eV, which is attributed to the lowest unoccupied molecular orbital (LUMO) peak. We followed a previously reported procedure[2] to determine the tilt angle and LUMO level (E_{LUMO}). Briefly, the tilt angle was determined from the ratio of the resonance peak intensity at NI and GI. The E_{LUMO} was estimated from the difference between the onset of the first resonance peak and C1*s* binding energy, corrected by exciton energy, which is 0.5 eV. The experimentally determined tilt angle and E_{LUMO} are shown in Table S1 and S2.

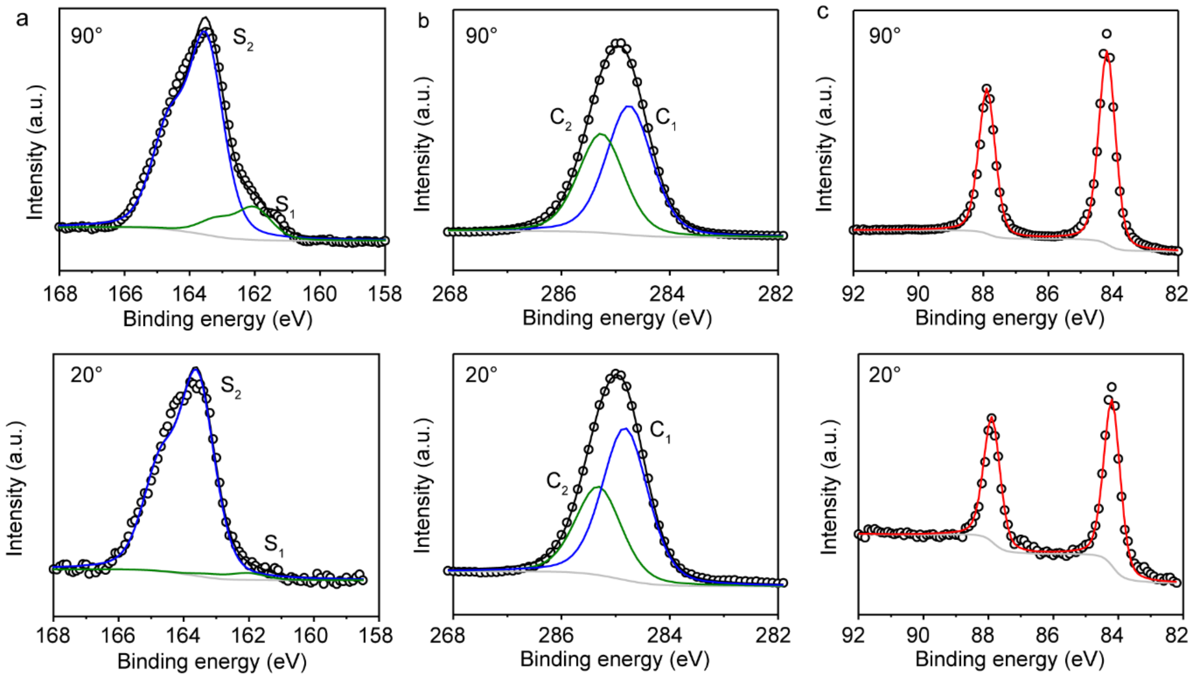

Figure S2. S 2*p* (a), C 1*s* (b) and Au 4*f* (c) spectra of S$(CH_2)_{11}$S-BTTF SAM on Au at take-off angle of 90° and 20°.

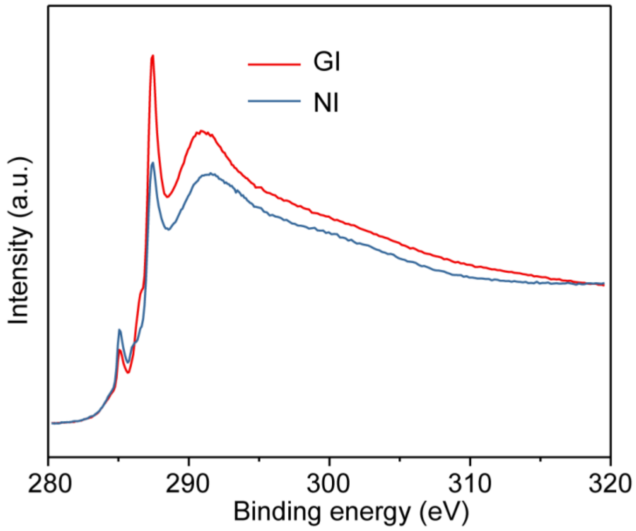

Figure S3. C K-edge NEXAFS spectra of S$(CH_2)_{11}$S-BTTF SAM on Au at NI and GI.

Ultraviolet photoelectron spectroscopy (UPS). To obtain the electronic properties of S$(CH_2)_{11}$S-BTTF SAM on Au, we performed UPS measurement by using an ESCALAB Mark 2 system (Omicron, Taunusstein, Germany) equipped with an Omicron (EA 125 U7) hemispherical electron spectrometer and He I source (21.2 eV). The work function (Φ),

highest occupied molecular orbital (HOMO) level (E_{HOMO}) and HOMO-1 level (E_{HOMO-1}) were determined from UPS spectra using previously reported procedures.[2] As shown in Figure S4, the red bars indicate the onset values of WF, HOMO and HOMO-1 (Table S2).

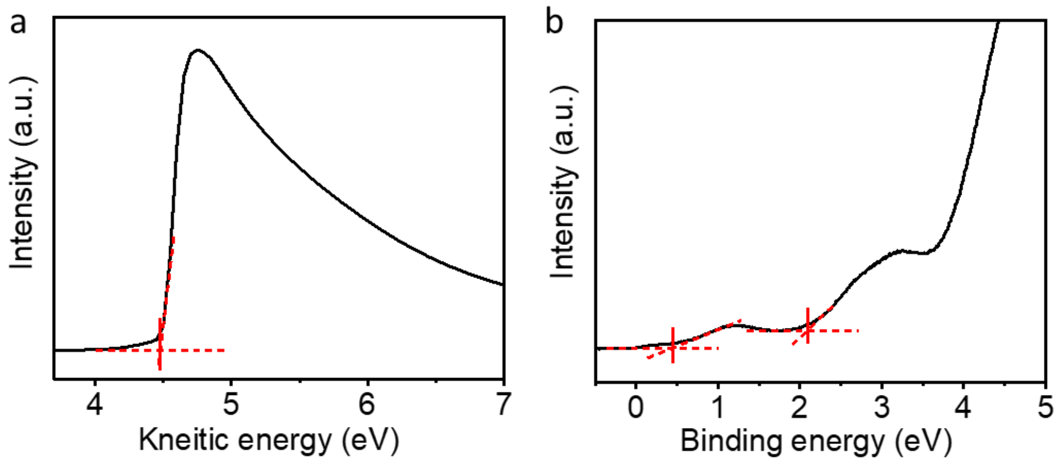

Figure S4. Secondary electron cutoff (SECO) (a) and valence band (b) spectra of $S(CH_2)_{11}S$-BTTF SAM on Au. The red bars indicate the onset of work function (Φ) and HOMO, HOMO-1 onset.

Section S3. Junction formation. The measurements of *J*(V,T) were carried out in a probe station (Lakeshore CRX-VF) under vacuum by using a molecular electronic device in a micropore on an ultra-smooth Au substrate with EGaIn stabilized in the through a channel of polydimethylsiloxane(PDMS) as the top electrode. We followed previously reported procedure to form micropore based junctions.[5] Briefly, the patterned Au (150 nm) was deposited on a Si wafer with its native SiO_2 layer by shadow mask deposition. Then the Si substrate was functionalized with 1H, 1H, 2H, 2H-perfluorooctyltrichlorosilane to reduce the interaction of the thermal adhesive with the template to enable the template-stripping. Next, we applied the thermal glue (EPOTEK 353ND) and glass support, cured the adhesive at 80 °C for 3 h and then we removed the glass/glue/Au composite from the template followed by deposition of 35 nm Al_2O_3 on the entire surface by atomic layer deposition at 90˚C. Next we

prepared micropore with a diameter of 10 µm on the Al_2O_3 surface using standard e-beam lithography and two-step etching processes as reported before[5]. The SAMs were formed by immersion of the substrates in the corresponding toluene solutions of the $(S(CH_2)_{11}S\text{-BTTF})_2$ as described in Section S1. Finally, the microchannels in PDMS were fabricated *via* previously reported procedure[5] and aligned over the array of micropores and filled with EGaIn.

S4. Statistical analysis and *J*(V,T) measurements. Since *J*(V,T) measurements are very time consuming, it is important to properly select representative devices. Therefore, for the *J*(V,T) measurements, we selected devices that had their *J*(V) characteristics within one log-standard deviation of the Gaussian log average *J*(V) curve ($<\log_{10}|J|>_G$ (V)) measured by cone-shaped tips of the EGaIn, as described in ref 6. Briefly, we collected 392 *J*(V) traces from 21 different junctions fabricated on 3 different substrates. The forward and backward traces for each individual curve were analyzed separately. The *J* values are log-normally distributed and therefore we determined the Gaussian log-average ($<\log_{10}|J|>_G$) values and the log-standard deviations at each measured voltage by fitting the values $\log_{10}|J|$ plotted in histograms to Gaussians (twice the square root of the number of data points was used as the bin number). As reported before[7], such log-normally distributed data sets have relatively large spread in the measured currents, but the trends are robust. In this work, the log-standard deviation of $\log_{10}|J|$ is 0.66 (see ref 6 for details), which means the spread in the currents $|J|$ for one-standard deviation is $|J| \times 4.57$ and $|J|$ / 4.57. This explains the seemingly large spread in the currents observed in the Arrhenius plots below although they all fall withing one log-standard, but we emphasize that the temperature dependency of the junctions is highly reproducible. We used individual *J*(V) traces at different temperatures to perform NDC

analysis as previously reported procedure[5, 8]. The 3D surfaces with color maps shown in the main text were constructed by using origin.

Figure S6 shows the data for junction 2 and Figure S7 shows the data for junction 3. Similar to the data set in the main text, a small negative E_a of -11 meV (Figure S6b for V = 1.5 V) and -14 meV (Figure S7b for V = 1.5 V) at low temperature is also observed, along with a bell shaped E_a vs. V curve (Section S5). It should be noted that at negative bias, E_a remains constant with E_a>200 meV (Figure S8c) for junction 2 which is due to the instability of the device likely caused by changes in the geometry of the junction induced by thermal contraction of the different components of the device (thermal expansion coefficient of PDMS = 3×10^{-4} K^{-1},[9] Au = 0.14×10^{-4} K^{-1},[10] Al_2O_3 = 0.054×10^{-4} K^{-1},[11] glass = 0.08×10^{-4} K^{-1},[12] GaO_x= 0.042×10^{-4} K^{-1},[13] and EGaIn=1.1×10^{-4} K^{-1} [14]) which is sometimes observed in our experiments. Junctions 3 also shows a clear increase of E_a at negative bias, but also here a noticeable offset from 0 eV is observable (Figure 8f). The J(V,T) data demonstrate that junction 3 has large a leakage current at negative bias, which results in a small R = 8 at 170 K. Likely, this leakage current is due to tunneling though a defect (see ref 15 for detailed discussions regarding causes of leakage currents) which explain the observed “background” E_a.

These two data sets confirm the robustness of our observations even in junctions where defects or changes in the junction geometry upon cooling of the devices are non-negligible.

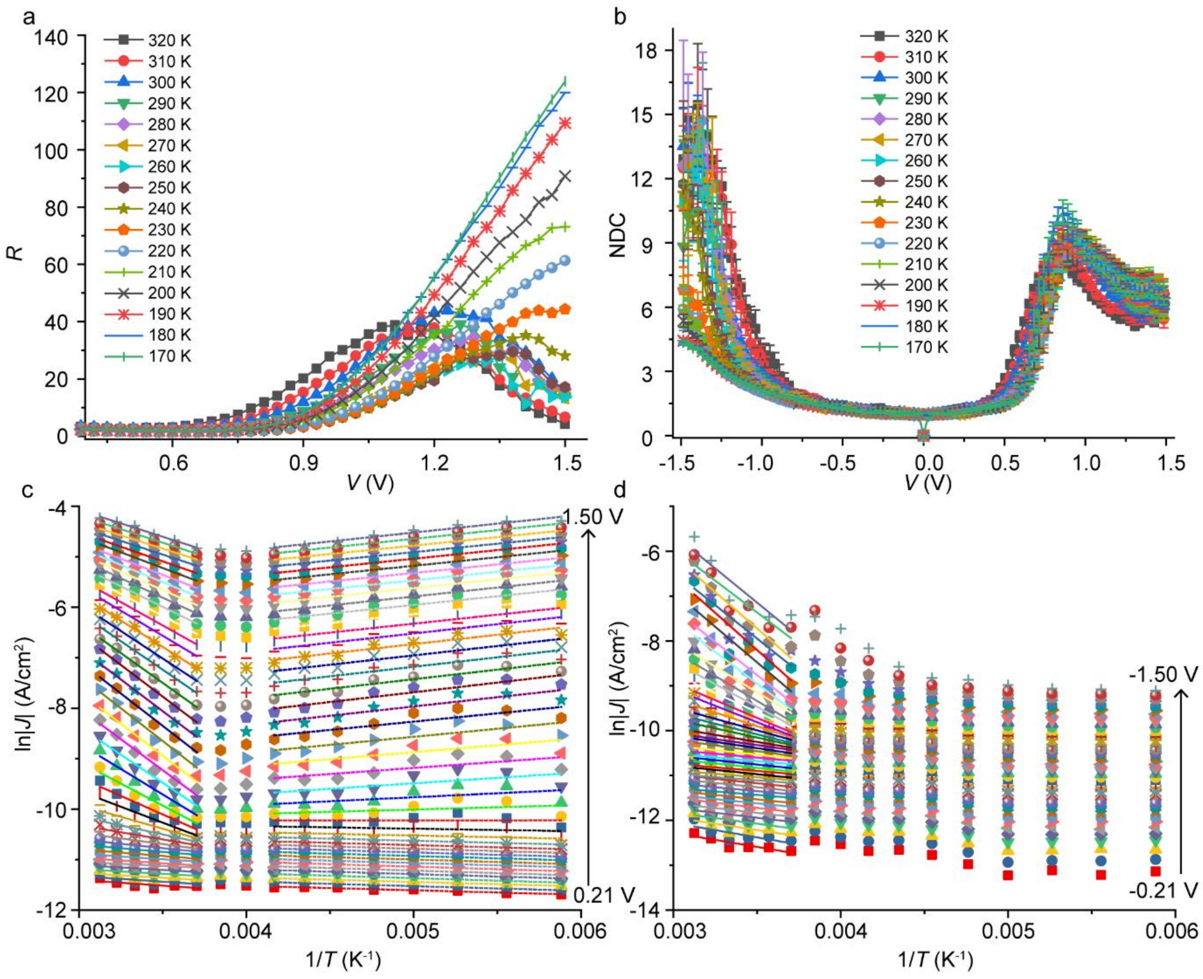

Figure S5. *R* vs. *V* over 170-320 K (a) and NDC plots (b) of the *J*(V,T) curves shown in Figure 2b. The error bars in panel b represent the standard deviation obtained from 3-5 traces measured at each temperature. Arrhenius plots from 0.21 to 1.5 V with 30 mV intervals (c) and from -0.21 to -1.5 V with -30 mV interval (d) for the data set in Figure 2b.

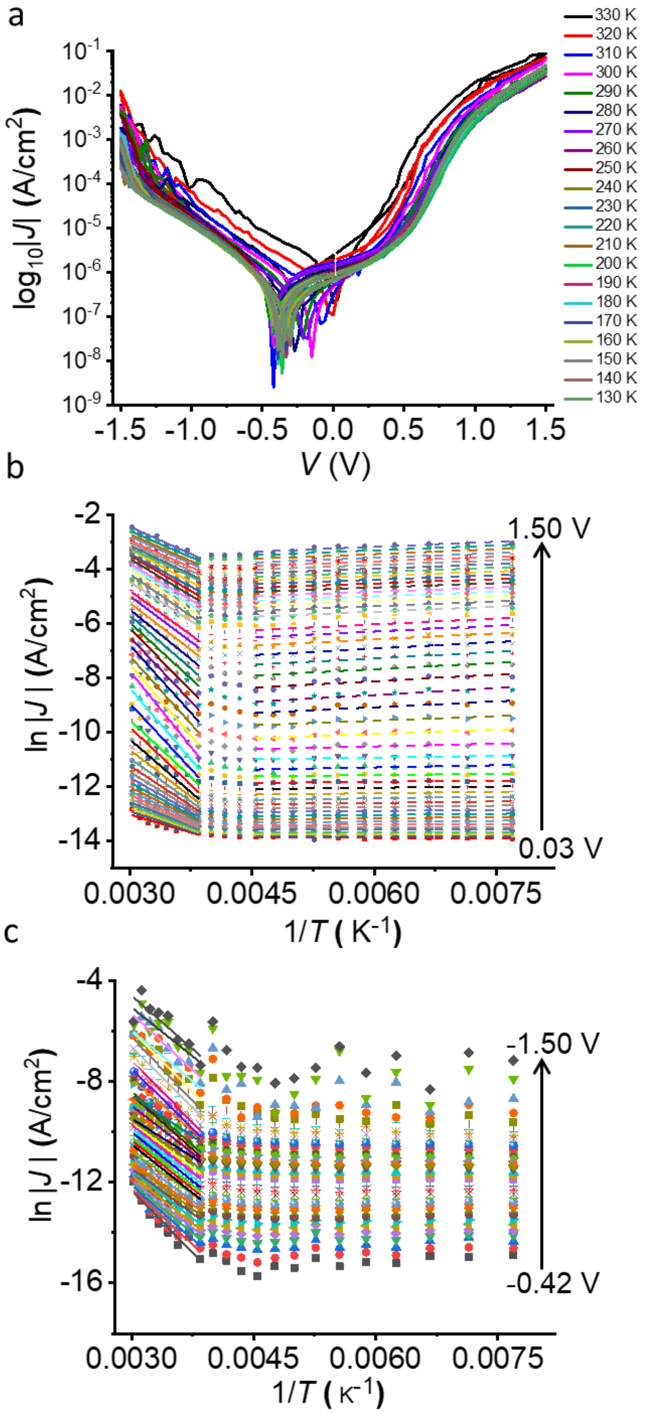

Figure S6. Data for junction 2. (a) Temperature dependent J(V) curves of Au-S$(CH_2)_{11}$S-BTTF//GaO_x/EGaIn junction measured over T = 130 - 330 K. (b) The corresponding Arrhenius plots for V from 0.03 V to 1.5 V at 30 mV intervals (a) and for V from -0.42 V to -1.5 V at -30 mV intervals (b) for J(V,T) curves. The solid and dashed lines are fits to Eq. 2.

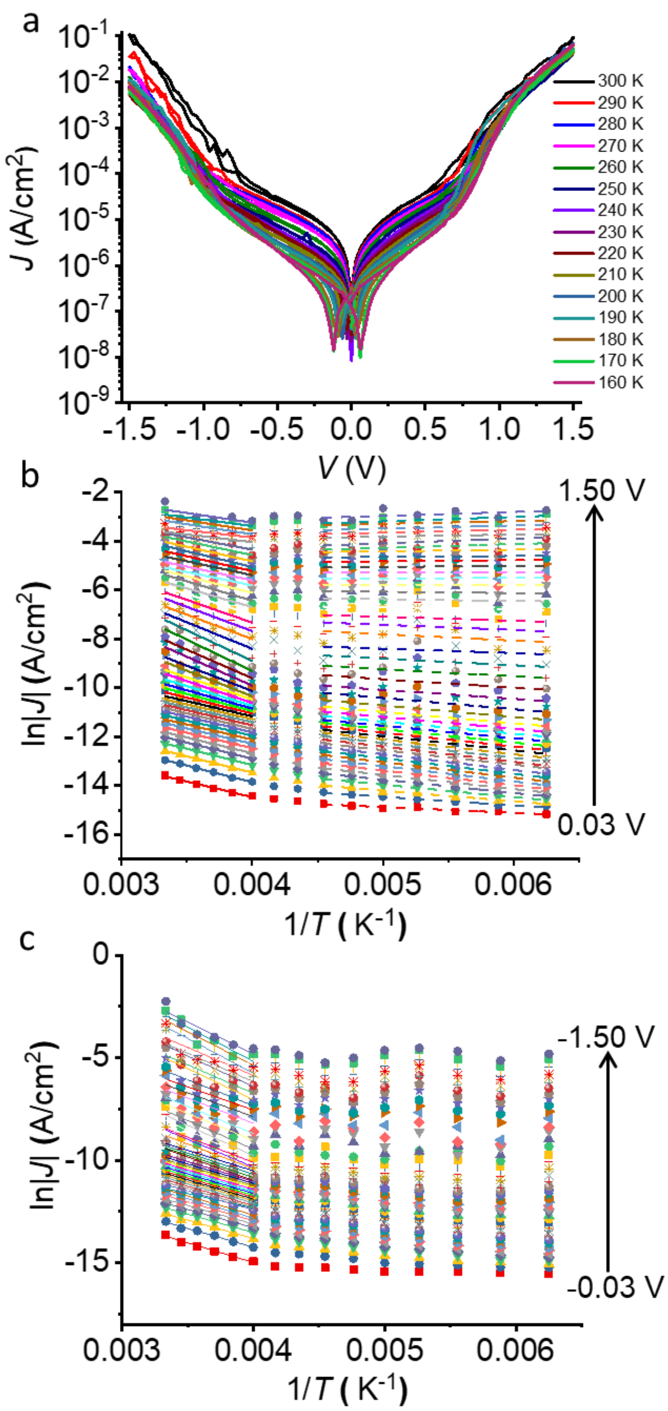

Figure S7. Data for junction 3. (a) Temperature-dependent *J*(V) curves of another Au-S$(CH_2)_{11}$S-BTTF//GaO_x/EGaIn junction measured over *T* = 300 -160 K at 10 K interval. (b) The corresponding Arrhenius plots for *V* from 0.03 V to 1.5 V at 30 mV intervals. (c) The corresponding Arrhenius plots for *V* from -0.03 V to -1.5 V at -30 mV intervals. The solid and dashed lines are fits to Eq. 2.

Section S5. Theoretical fitting. The calculated activation energy as a function of the internal coupling gate potentials (orbital gating) for junction 2 and junction 3 are shown in Figure S8a and d. These were calculated using the Migliore et al.[16] model discussed in the main text. The inset in Figure 8b and e show the calculated charge distribution for the data sets using Eq.5 in the main text. The parameters for all of the fits are shown in Table S3.

Table S3. Parameters for all of the fits

Data Set	Data sets for Junction 2	Data sets for Junction 3
Energy Difference ($\Delta \boldsymbol{E_0}$)	0.89 eV	0.459 eV
Reorganization Energy ($\boldsymbol{\lambda}$)	1.22 eV	0.822 eV
Coupling Capacitance ($\boldsymbol{C_C^*}$)	1.45 x 10^{-19} F	0.923 x 10^{-19} F
Center of I-V Curve (ε_Q)	0.51	0.81
Width of I-V Curve (σ_Q)	0.3	0.266

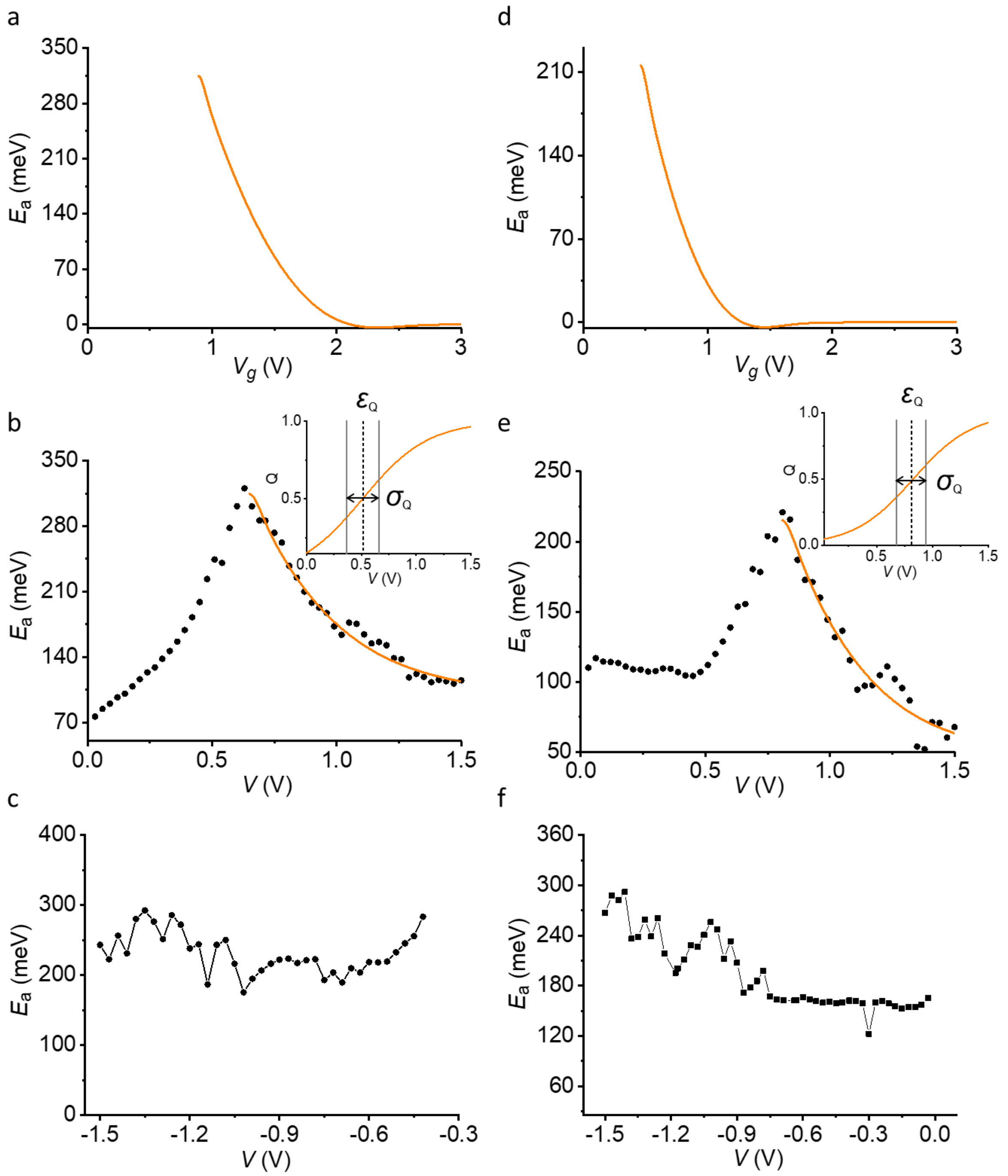

Figure S8. Calculated activation energy for the data sets in Figure S6b (a) and S7b (d) (solid line) using Eq.5 in the main text. The activation energy E_a determined from the Arrhenius plots in Figure S6b (b) and S7b (e) as a function of positively applied bias (black symbols) and the fitted curve (orange lines). The insets show the charge distribution for the data sets using Eq.5 in the main text. The activation energy E_a determined from the Arrhenius plots in Figure S6c (c) and S7c (f) vs. negative applied bias.

References

[1] L. Yuan, L. Jiang, C. A. Nijhuis, *Adv. Funct. Mater.* **2018**, *28*, 1801710.

[2] L. Yuan, R. Breuer, L. Jiang, M. Schmittel, C. A. Nijhuis, *Nano Lett.* **2015**, *15*, 5506-5512.

[3] W. Du, Y. Han, H. Hu, H.-S. Chu, H. V. Annadata, T. Wang, N. Tomczak, C. A. Nijhuis, *Nano Lett.* **2019**, *19*, 4634-4640.

[4] M. Souto, V. Díez-Cabanes, L. Yuan, A. R. Kyvik, I. Ratera, C. A. Nijhuis, J. Cornil, J. Veciana, *Phys. Chem. Chem. Phys.* **2018**, *20*, 25638-25647.

[5] S. K. Karuppannan, H. Hongting, C. Troadec, A. Vilan, C. A. Nijhuis, *Adv. Funct. Mater.* **2019**, *29*, 1904452.

[6] Y. Han, M. S. Maglione, V. Diez Cabanes, J. Casado-Montenegro, X. Yu, S. K. Karuppannan, Z. Zhang, N. Crivillers, M. Mas-Torrent, C. Rovira, J. Cornil, J. Veciana, C. A. Nijhuis, *ACS Appl. Mater. Interfaces* **2020**, *12*, 55044-55055.

[7] W. F. Reus, C. A. Nijhuis, J. R. Barber, M. M. Thuo, S. Tricard, G. M. Whitesides, *J. Phys. Chem. C* **2012**, *116*, 6714-6733.

[8] A. Vilan, *Phys. Chem. Chem. Phys.* **2017**, *19*, 27166-27172.

[9] S. Lee, H. Tung, W. Chen, W. Fang, *IEEE Photon. Technol. Lett.* **2006**, *18*, 2191-2193.

[10] D. R. Lide, *Handbook of Chemistry and Physics*, CRC Press, Inc.: Boca Raton, FL, **1996**.

[11] P. Auerkari, *Mechanical and Physical Properties of Engineering Alumina Ceramics*, VTT Offsetpaino, Espoo, Finland, **1996**.

[12] Y. Menke, V. Peltier-Baron, S. Hampshire, *J. Non-Cryst. Solids.* **2000**, *276*, 145-150.

[13] M. Yamaga, E. G. Víllora, K. Shimamura, N. Ichinose, M. Honda, *Phys. Rev. B.* **2003**, *68*, 155207.

[14] J. N. Koster, *Cryst. Res. Technol.* **1999**, *34*, 1129-1140.

[15] D. Thompson, C. A. Nijhuis, *Acc. Chem. Res.* **2016**, *49*, 2061-2069.

[16] A. Migliore, P. Schiff, A. Nitzan, *Phys. Chem. Chem. Phys.* **2012**, *14*, 13746-13753.
